# Brain Transcriptome-Wide Screen for HIV-1 Nef Protein Interaction Partners Reveals Various Membrane-Associated Proteins

**DOI:** 10.1371/journal.pone.0051578

**Published:** 2012-12-17

**Authors:** Ellen C. Kammula, Jessica Mötter, Alexandra Gorgels, Esther Jonas, Silke Hoffmann, Dieter Willbold

**Affiliations:** 1 Institute of Complex Systems, ICS-6: Structural Biochemistry, Forschungszentrum Jülich, Jülich, Germany; 2 Institute of Physical Biology, Heinrich-Heine University Düsseldorf, Düsseldorf, Germany; National Institute of Health, United States of America

## Abstract

HIV-1 Nef protein contributes essentially to the pathology of AIDS by a variety of protein-protein-interactions within the host cell. The versatile functionality of Nef is partially attributed to different conformational states and posttranslational modifications, such as myristoylation. Up to now, many interaction partners of Nef have been identified using classical yeast two-hybrid screens. Such screens rely on transcriptional activation of reporter genes in the nucleus to detect interactions. Thus, the identification of Nef interaction partners that are integral membrane proteins, membrane-associated proteins or other proteins that do not translocate into the nucleus is hampered. In the present study, a split-ubiquitin based yeast two-hybrid screen was used to identify novel membrane-localized interaction partners of Nef. More than 80% of the hereby identified interaction partners of Nef are transmembrane proteins. The identified hits are GPM6B, GPM6A, BAP31, TSPAN7, CYB5B, CD320/TCblR, VSIG4, PMEPA1, OCIAD1, ITGB1, CHN1, PH4, CLDN10, HSPA9, APR-3, PEBP1 and B3GNT, which are involved in diverse cellular processes like signaling, apoptosis, neurogenesis, cell adhesion and protein trafficking or quality control. For a subfraction of the hereby identified proteins we present data supporting their direct interaction with HIV-1 Nef. We discuss the results with respect to many phenotypes observed in HIV infected cells and patients. The identified Nef interaction partners may help to further elucidate the molecular basis of HIV-related diseases.

## Introduction

Human immunodeficiency virus type 1 (HIV-1) primarily infects CD4^+^ T cells and cells of the monocyte-macrophage lineage. In addition to immune deficiency, HIV-1 is the direct source for a number of neurological symptoms, suggesting that HIV-1 is able to enter the central nervous system (CNS) and cause neurocognitive impairment, especially at later stages of the infection. The HIV-1 Nef protein is an accessory protein that plays an important role in the infectivity, persistence and pathology of the virus. Its importance in the progression of AIDS is evident, since it is known that deletion or absence of Nef attenuates the symptoms in HIV patients [1]. The downmodulation of cell surface levels of CD4 as well as the downmodulation of major histocompatibility class I (MHC I) molecules, the mediation of cellular signaling and activation, and the enhancement of viral particle infectivity are the four most thoroughly documented Nef activities that affect immune cells and have together with other aspects been extensively reviewed elsewhere [2]–[5]. The number of cell surface receptors modulated by Nef is steadily increasing [6], but it is still not clear how any of these interactions contribute to HIV pathogenesis.

Several attempts have been made to identify host cell proteins that interact with Nef and to elucidate Nef mediated pathogenic effects. Up to now however, all published yeast two-hybrid (Y2H) screens with Nef as a bait were performed with conventional protocols using classical Y2H screens that rely on transcriptional activation of reporter genes in the nucleus [7]–[13]. Thus, the detection of interaction partners that are integral membrane proteins, membrane-associated proteins or other proteins that do not translocate into the nucleus was impeded in those studies.

Because Nef is posttranslationally myristoylated and thus is at least transiently localized to membranes, we set out to employ a screening procedure that is potentially able to identify membrane proteins.

## Results

We performed a split-ubiquitin based membrane-associated Y2H screen using a membrane-anchored Nef as a bait to facilitate the identification of further Nef binding host cell proteins, which are integrated in or associated to membranes. Because we were especially interested in HIV-induced processes in the brain, we used a human brain cDNA library to screen for Nef interacting partners.

### Design and Results of the Yeast Two-hybrid Screen

**Figure 1:**
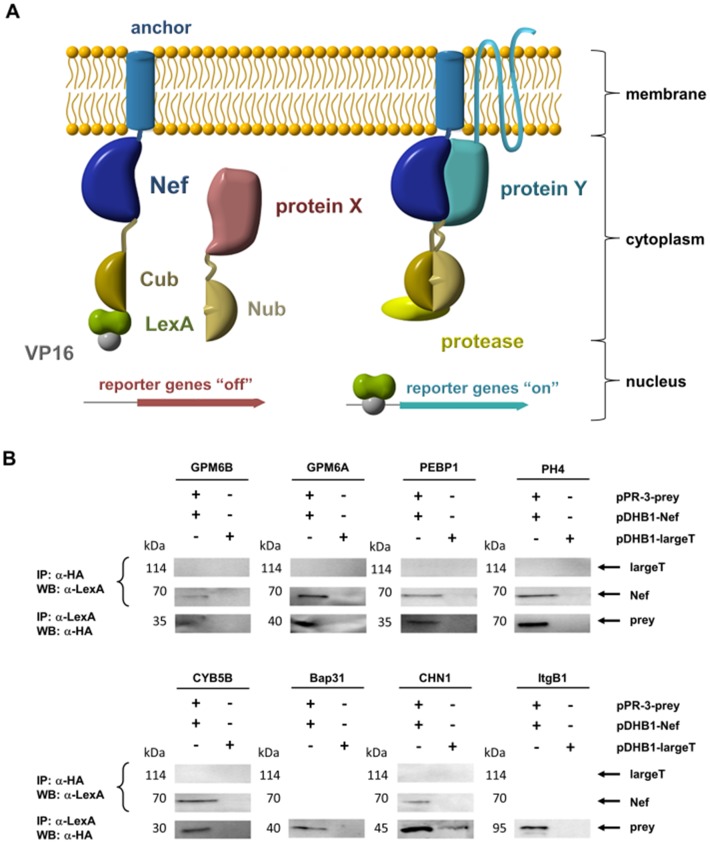
Schematic of the Y2H screen and coimmunoprecipitation of selected preys show interaction with Nef. A. Schematic presentation of the split-ubiquitin Y2H screen with membrane-anchored Nef as bait. Wild type Nef myristoylation is replaced by the Ost4p transmembrane anchor and amino acids 39–76 of yeast ubiquitin (Cub) linked to the LexA-VP16 transcription factor carboxy-terminally to Nef. A human adult brain cDNA library (Dualsystems Biotech AG) cloned in pPR3-N expressing the cDNAs as fusions carboxy-terminally of amino acids 1–38 of yeast ubiquitin (Nub) and an HA-tag. Upon binding of the bait and prey both parts of the ubiquitin come together and are cleaved by the protease to activate reporter genes. If no interaction with the Nef bait protein is possible, the ubiquitin subunits stay apart and no reporter genes in the nucleus are turned on. B. Coimmunoprecipitation (CoIP) of Nef and preys. Yeast cell lysate proteins from different transfected cultures as well as a non-transfected control (NMY51) as indicated at the top were immunoprecipitated (IP) either with anti-HA or anti-LexA antibodies. For negative controls, cells were alternatively transfected with a bait expression vector coding for the SV40 large T antigen (largeT) instead of Nef. The resulting immunoprecipitates were electrophoretically separated, blotted (WB) on a PVDF-membrane as indicated at the right and detected with anti-Myc or anti-HA antibodies. The approximate molecular weights of the proteins are shown. Two additional experiments gave similar results.

To account for Nef’s increased attraction to membranes upon its posttranslational myristoylation, we used full length Nef fused to an Ost4p transmembrane anchor at its N-terminus. At the C-terminal end, this bait contained the C-terminal part of ubiquitin (Cub) linked to the transcription factor LexA-VP16. For a lot of its activities, Nef requires dimerization, which is mediated by interactions between residues located in the Nef core region [14]. Fusion of Nef to Ost4p does not restrict dimerization in any way. The schematic of the Y2H system with the resulting bait plasmid (pDHB1-Nef) is shown in Figure 1. Preys were from a purchased human adult brain cDNA library, which covered approx. 1.75×10^6^ independent clones and was linked to the N-terminal part of ubiquitin (Nub). The expression of the membrane-localized Nef-bait was verified by Western blotting of cell extracts using a mouse monoclonal antibody directed against the LexA domain (data not shown).

The screen was carried out as described in the Methods part. Altogether, 58 different host cell proteins were obtained as hits from the initial screen. It is well known that Y2H screens, like any genetic selection system, are prone to produce a certain number of false positive results. Such hits would show a His^+^/Ade^+^/lacZ^+^ phenotype independent of a true interaction between the bait and the prey. In order to eliminate such false positive clones, the respective pPR3 vector-DNAs of each of the 58 hits were tested for the His^+^/Ade^+^/lacZ^+^ phenotype. Approximately half of the initially identified hits showed β-galactosidase activity when coexpressed with the Nef-bait but not when coexpressed with the large T control bait. Only those were further considered to be Nef dependent positive hits. The results of the positive hits are summarized in Table 1 listing the names and their hit frequencies. Altogether, we identified the following proteins, although with very different frequencies: the neuronal membrane glycoprotein M6B (GPM6B), the B-cell receptor-associated protein 31 (BAP31), the glycoprotein M6A (GPM6A), Tetraspanin 7 (TSPAN7), Cytochrome B5 type B (CYB5B), the receptor for transcobalamin-bound vitamin B12 (CD320/TCblR) and Vitamin K epoxide reductase complex, subunit 1-like protein 1 (VKORC1L1), V-set and immunoglobulin domain containing 4 (VSIG4), Transmembrane prostate androgen-induced protein (PMEPA1), OCIA domain containing 1 (OCIAD1), Integrin beta-1 (ITGB1), Chimerin 1 (CHN1), Hypoxia-inducible factor (HIF) prolyl 4-hydroxylase (PH4), Claudin 10 (CLDN10), Heat shock 70 kDa protein 9/mortalin (HSPA9), Apoptosis-related protein 3 (APR-3), Solute carrier family 31 (copper transporter), member 2 (SLC31A2), Phosphatidylethanolamine binding protein 1 (PEBP1), Probable cation-transporting ATPase 13A2 (ATP13A2), V-type proton ATPase subunit S1 (ATP6AP1) and N-acetyllactosaminide beta-1,3-N-acetylglucosaminyltransferase (B3GNT1). Detailed information (gene ID, subcellular localization, function) for each protein is given in the Table S1. Of those, VKORC1L1, SLC31A2, ATP13A2 and ATP6AP1 were not further considered, because they have been reported to appear unspecifically as false-positive hits based on the components of the DUALhunter system itself (for reference see http://www.dualsystems.com).

**Table 1:** Analysis of the bait dependency test.

protein	N° of hits	pDHB1-Nef			pDHB1-LargeT			CoIP	
		SD –leu -trp	SD -leu -trp -his -ade	β-gal	SD -leu -trp	SD -leu -trp –his -ade	β-gal	IP:α-LexA WB:α-HA	IP: α-HA WB:α-LexA
GPM6B	23	5	5	5	5	2	0	+	+
BAP31	10	5	5	5	5	0	0	-	+
CD320	1	6	6	6	6	2	0	nd	nd
CYB5B	2	8	8	7	5	2	0	+	+
GPM6A	2	8	8	8	5	0	0	+	+
TSPAN7	2	5	5	5	5	0	0	nd	nd
APR-3	1	6	6	6	6	4	0	nd	nd
B3GNT1	1	5	5	5	5	0	0	nd	nd
CHN1	1	6	6	6	6	1	0	+	+
CLDN10	1	6	6	5	6	5	0	nd	nd
HSPA9	1	6	6	6	6	4	0	nd	nd
ITGB1	1	6	6	6	6	0	0	-	+
OCIAD1	1	6	6	6	6	0	0	nd	nd
PEBP1	1	3	3	3	5	2	0	+	+
PH4	1	5	5	5	5	0	0	+	+
PMEPA1	1	6	6	5	6	0	0	nd	nd
VSIG4	1	6	6	6	6	0	0	nd	nd
*VKORC1L1*	*2*	*2*	*2*	*2*	*5*	*0*	*0*	*nd*	*nd*
*ATP13A2*	*1*	*6*	*6*	*6*	*6*	*5*	*0*	*nd*	*nd*
*ATP6AP1*	*1*	*6*	*6*	*6*	*6*	*1*	*0*	*nd*	*nd*
*SLC31A2*	*1*	*6*	*6*	*6*	*6*	*4*	*0*	*nd*	*nd*

Bait dependency test for growth on minimal media (His^−/^Ade^−/^lacZ^−^) of the positive preys coexpressed with pDHB1-Nef or pDHB1-LargeT (negative control) and summarized CoIP results (last column). Only hits that passed the Nef dependency test are listed. The table is sorted by the number of hits, which is given beside the protein name. Putative Nef-interacting proteins prone to be false-positive interactors based on the components of the Y2H system itself (according to the Dualsystems support page) are written in italic.

More than 80% of the identified proteins are localized in membranes (17 out of 21) with membrane topologies containing at least one transmembrane domain (TM) and spanning up to 12 TMs.

Yeast cells cotransfected with the prey vectors from eight hits (GPM6B, GPM6A, BAP31, CHN1, CYB5B, ITGB1, PEB1, PH4) and either pDHB1-Nef or pDHB1-largeT were directly subjected to coimmunoprecipitation (CoIP) experiments (Figure 1B). As summarized in Table 1, these immunoprecipitations (IP) experiments confirmed the existence of precipitable immune complexes including Nef and each of the eight hit proteins tested in yeast cell lysates. Notably, detection of prey protein complexes with the SV40 large T antigen as negative control bait was weak for CHN1 and not visible for all other samples tested. This suggests that the detected Nef-prey complexes represent specific interactions and are not caused by unspecific protein aggregation. In case of BAP31 and ITGB1, a CoIP of yeast cell lysates could be demonstrated only in one direction (IP with anti-Lex and WB with anti-HA).

For a selection of the identified Nef interacting proteins we have carried out colocalization studies in Cos-7 cells, as described in the following.

### Colocalization by Confocal Microscopy Studies on Selected Hits

**Figure 2:**
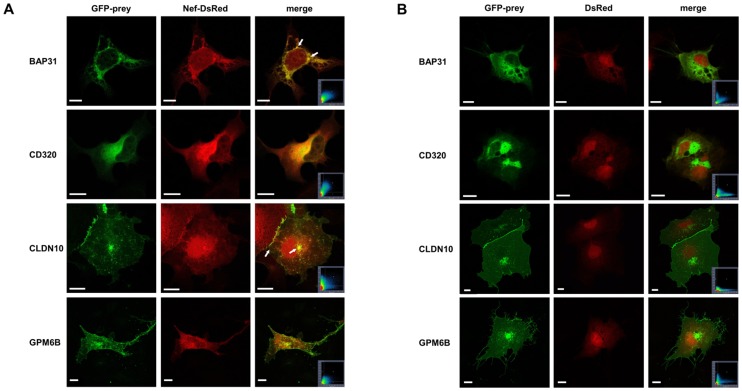
Analysis of subcellular localization of BAP31, CD320/TCblR, CLDN10 and GPM6B with and without Nef. A. Confocal microscopy analysis of Cos-7 cells coexpressing GFP fusions of BAP31, CD320/TCblR, CLDN10 or GPM6B with Nef-DsRed. Cos-7 cells were transiently cotransfected with pNef-DsRed and pGFP-BAP31, pGFP-CD320/TCblR, pGFP-CLDN10 or pGFP-GPM6B and fixed 24 h posttransfection. Single images from the red (Nef-DsRed) and green (GFP-prey) channels were overlaid in the merged image. Yellow regions represent colocalization, details are given in the text. Arrows point out BAP31 accumulations and distinct areas of CLDN10 and Nef overlay. Scale bar: 10 µm. B. Negative controls for the colocalization images in Figure A. Confocal microscopy analysis of Cos-7 cells coexpressing GFP fusions of BAP31, CD320/TCblR, CLDN10 or GPM6B with DsRed. Cos-7 cells were transiently cotransfected with pDsRed and pGFP-BAP31, pGFP-CD320/TCblR, pGFP-CLDN10 or pGFP-GPM6B and fixed 24 h posttransfection. Single images from the red (DsRed) and green (GFP-prey) channels were overlaid in the merged image. The respective scatter grams, as well as the images and scatter grams from cotransfections with the pDsRed control vector are given.

To further evaluate the significance of a subfraction of the hits with regard to their physiological relevance on cellular level, we analyzed their cellular distribution in absence and presence of Nef in eukaryotic cells. Such experiments were performed with BAP31, CD320/TCblR, CLDN10, and GPM6B. For this, pGFP-BAP31, pGFP-CD320, pGFP-CLDN10 and pGFP-GPM6B expression plasmids, a pNef-DsRed expression plasmid and a pDsRed (Clontech) control vector were constructed and used for transient transfection of Cos-7 cells as described in the Methods chapter. Figure 2A summarizes the results obtained from cells that have been cotransfected with the pGFP-host protein fusions together with pNef-DsRed.

A representative result of the coexpression of GFP-BAP31 and Nef-DsRed is shown in the top panel of Figure 2 and in the merged image colocalization of BAP31 and Nef mainly along membranes of the nucleus or ER/Golgi structures was seen. Notably, the strongest colocalization occurs at the perinuclear BAP31 accumulations (arrows). Cos-7 cells expressing GFP-BAP31 alone also demonstrate a subcellular localization of the BAP31 fusion protein adjacent to the nucleus and along ER/Golgi structures. Depending on the area of the focal plane, some cells showed prominent perinuclear accumulation (data not shown). These accumulations are consistent with previous findings as made by Wakana et al. [15]. No colocalization is visible at the plasma membrane and inside the cytoplasm itself, where only Nef-DsRed can be imaged.

The same experiments were carried out for CD320/TCblR, CLDN10 and GPM6B. For CD320/TCblR, the strongest fluorescence overlap was observed at the perinuclear region. CLDN10, a protein in tight junctions, was visible at the border of two adjacent cells, close to the nucleus and at some punctuate likely vesicular structures inside the cell. Remarkably, when overexpressing CLDN10 we observed that Nef clearly is additionally localized in the tight junction region and at the vesicular CLDN10 positive structures (arrows).

When GPM6B was cotransfected with Nef, colocalization of the two fusion proteins at the perinuclear region and in most of the vesicular accumulations was observed. To rule out an influence of the fluorescent protein fusions, each GFP-host protein expression construct was cotransfected with an empty pDsRed vector as control (Figure 2B). These negative controls showed no or hardly any overlap of the green and red fluorescence channels. Thus, an effect of the fusion proteins can be very likely excluded and all observed colocalizations are due to the close proximity or the direct interaction of Nef with the analyzed proteins.

**Figure 3:**
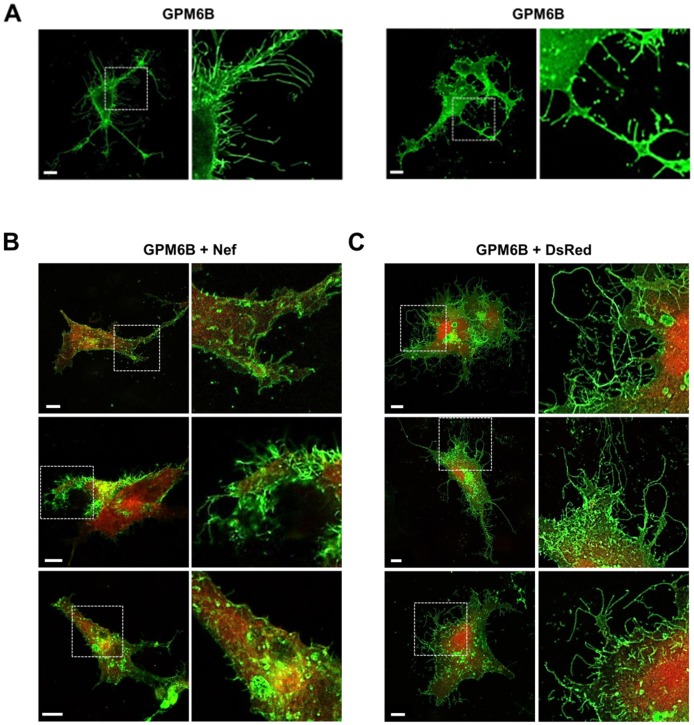
Analysis of GPM6B-induced outgrowth in Cos-7 cells. Cells were treated as described in Figure 2. For enhanced visualization of the membrane extensions, magnifications of the boxed areas are depicted to the right of each picture. Representative images of cells transfected with pGFP-GPM6B without (A) or with pNef-DsRed (B) or pDsRed (C) cotransfection are shown. Scale bar: 10 µm.

Notably, transiently transfected Cos-7 cells displayed a different morphology when expressing GFP-GPM6B. Their plasma membrane was untypically frayed and structures that are reminiscent of neurite-like outgrowths or filopodia, which recently had been described for Cos-7 cells by others [16], [17], could be observed. These structures showed high levels of GPM6B (Figure 3A). Interestingly, the presence of Nef affected the extent to which the membrane extensions occurred. In general, less and shorter outgrowths were observed when Nef was present in the cells in addition to GPM6B (Figure 3B). Cos-7 cells cotransfected with pGFP-GPM6B and the empty control vector (pDsRed) did not show significant changes in the length of the extensions compared to cells transfected only with pGFP-GPM6B. This suggests that the GPM6B-mediated formation of membrane extensions is negatively influence by the Nef protein (Figure 3C).

The distributions of the respective GFP-host protein expression constructs were compared with the expression patterns of the respective endogenous proteins described by others (e.g. [15], [18]), or that were obtained in our laboratory by direct immunostaining of fixed Cos-7 cells using commercially available primary antibodies. In the case of CD320/TCblR, which is endogenously expressed only at very low levels in Cos-7 (as well as in most other cell lines), the immunostaining of endogenous CD320/TCblR was not possible. For GFP-BAP31 and GFP-CLDN10, no big differences could be observed compared to their endogenous counterparts (data not shown).

### *In vitro* Binding Studies of GPM6B with Nef

**Figure 4:**
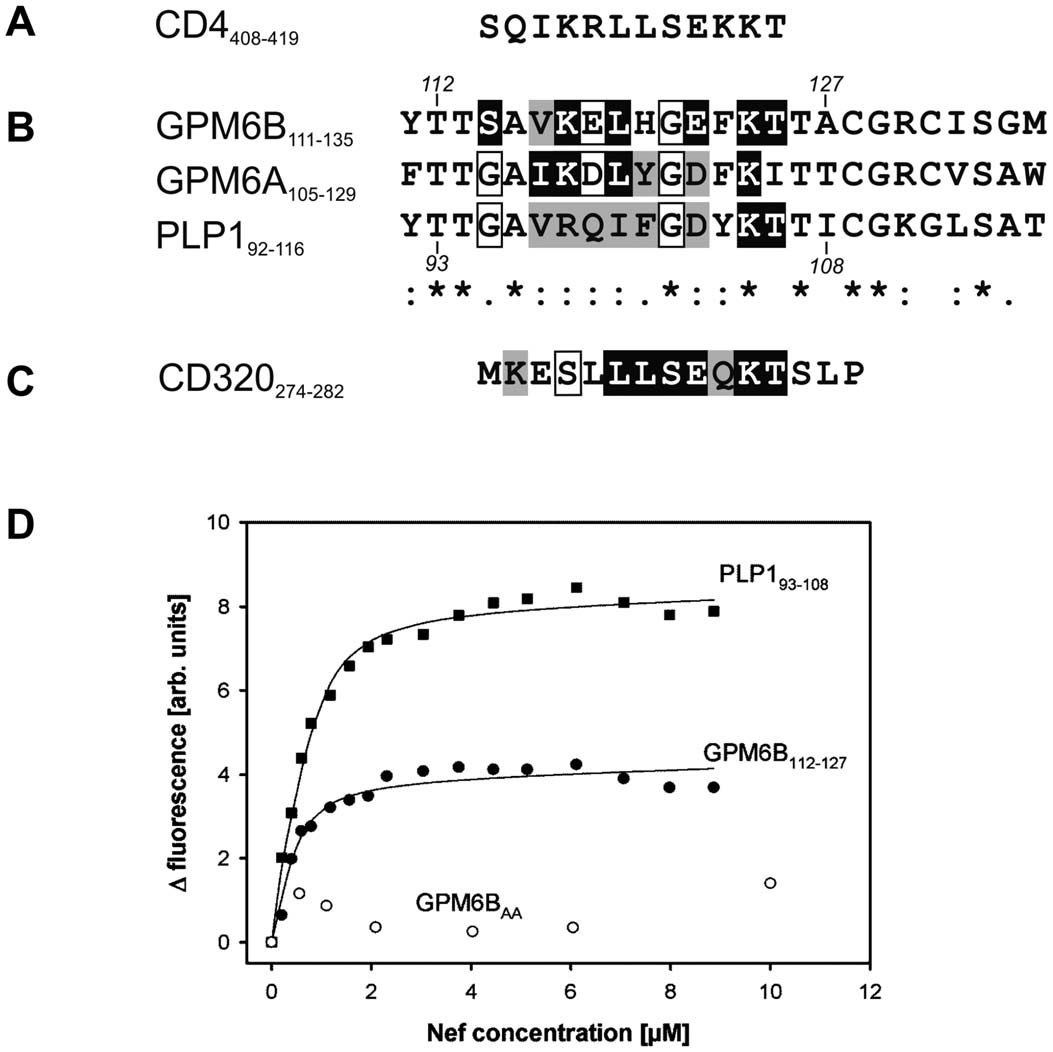
Sequence analysis of Nef binding site and *in vitro* binding studies of GPM6B and Nef. A. Amino acid sequence of the Nef binding motif of CD4. B. Clustal W [70] based alignment of the cytoplasmic loops of GPM6B, GPM6A and PLP1. Note, for GPM6B, GPM6A and PLP-DM20/PLP1 isoform 2, the sequences of the complete cytoplasmic loop regions are shown. Additionally, positions of the indicated proteolipid proteins with homologies to a sequence region in the cytoplasmic tail of T-cell surface glycoprotein CD4 (shown at the top) that includes the core Nef binding motif of CD4 [20], [21] are highlighted. Positions identical to the respective CD4 residue are shaded black, those with high similarity are shaded grey and those with lower similarity are boxed. The indicated residue numbers show the beginning and the end of the amino-terminally fluoresceinylated GPM6B and PLP1 peptides used for the Nef binding studies shown in D. Numbering of GPM6B is based on UniProtKB entry Q13491-1 throughout this figure. C. Sequence of the cytoplasmatic portion of CD320/TCblR, homologies to CD4 indicated as described above. D. Fluorescence titration of 0.5 µM of fluoresceinyl-labeled peptides, GPM6B_112–127_, GPM6B_AA_, or PLP_193–108_ with recombinant HIV-1SF2 Nef_2–210_ protein (prepared as described in [68]). The fluorescence signals are shown as a function of the Nef_2–210_ protein concentration. Values result from the fluorescence of the peptides in the presence of the indicated concentration of Nef_2–210_ in comparison with a buffer control titration. Assuming a simple bimolar interaction between the peptide and Nef_2–210_, the data were described by a model based solely on the law of mass action which accounts for ligand depletion [71]. Nonlinear curve fitting of the model to the fluorescence data (lines) yielded dissociation constants of 0.64±0.06 µM for GPM6B_112–127_ and of 0.71±0.03 µM for PLP_193–108_.

A brief look at the GPM6B amino acid sequence of the cytoplasmic loops revealed a striking similarity to residues 408 to 419 inside the cytoplasmic domain of the T-cell co-receptor CD4. This region is known to be necessary and sufficient for downregulation of CD4 by Nef (e.g. reviewed in [5], [6]) and also its direct binding to Nef is well characterized ([19]–[21]). Thus, we investigated, whether any of the other proteins identified from the Y2H-screen show similarities to already known Nef interaction motifs. Comparison of residues 114 to 125 of the GPM6B cytoplasmic loop (Q13491-1) and 108–119 of GPM6A with the sequence of the Nef binding motif of CD4 (amino acids 408 to 419 of the mature protein chain; Figure 4A) resulted in striking sequence similarities. In this region, GPM6B and CD4 sequences show a 50% identity and 25% similarity by only three out of twelve residues that do not fit the alignment. The alignment of the respective regions of GPM6A and PLP1 with this CD4 region also showed striking similarities (Figure 4B). This suggests that the conserved cytoplasmic loop region of GPM6B and GPM6A might contain a Nef binding site similar to that of CD4. This region is notably also conserved in the myelin proteolipid protein (PLP1), which is the prototype protein of the PLP family. As shown in Figure 4D, fluorescence titrations using purified Nef protein and synthetic FITC-labeled peptides derived from the cytoplasmic parts of GPM6B or PLP1 respectively, verified a direct and rather robust interaction between the molecules with K*_D_* values ranging below 1 µM. Mutations in CD4, in which leucines 413 and 414 were replaced with alanines, renders CD4 refractory to Nef-induced downregulation [22]–[24] and at least *in vitro* drastically reduces the binding affinity of synthetic CD4 peptides to full-length Nef protein [20], [21]. If this is the same binding determinant as the previously identified and well known Nef binding site of CD4, one may expect that the residues in GPM6B that correspond to the dileucine-motif also play an important role. Therefore, we also analyzed a peptide variant (GPM6B_AA_) in which the dileucine-motif corresponding residues leucine 119 and histidine 120 of GPM6B were replaced by alanines. The relative fluorescence of FITC-labeled GPM6B_AA_ was virtually independent of Nef concentration, suggesting that it did not bind to Nef (Figure 4D, open circles). The mutation of leucine 119 and histidine 120 to alanines drastically reduced the affinity of the examined GPM6B peptide to Nef. This clearly shows that both residues contribute to Nef-GPM6B binding. It underlines the similarities between the Nef binding sites in CD4 and PLP-like proteins. Very interestingly, also in CD320/TCblR a region with high similarity to the Nef binding site of CD4 could be identified inside its short cytoplasmic portion (Figure 4C). In this region, CD320/TCblR and CD4 sequences show also 50% identity and 25% similarity.

## Discussion

**Figure 5:**
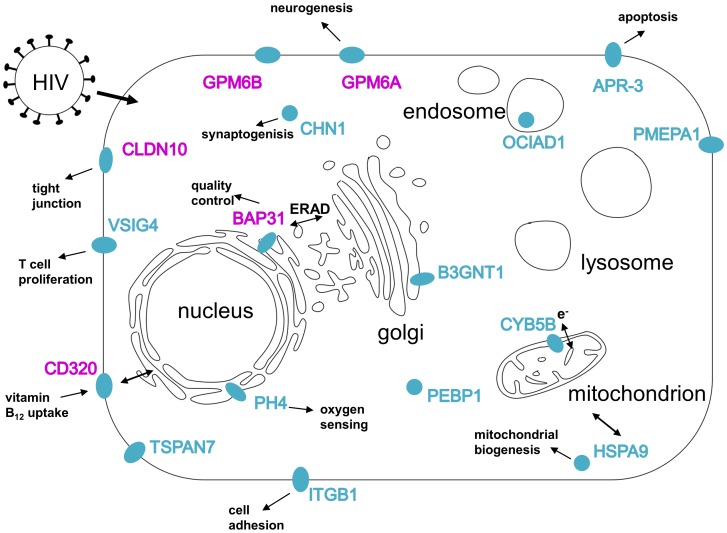
Subcellular localization of the identified hits within a model cell. Overview of the subcellular localization of the interaction partners of Nef identified via the split-ubiquitin based Y2H system based on the data given in Table S1. Marked in magenta are the proteins studied in more detail.

We successfully performed a membrane-associated Y2H screen with a membrane-anchored HIV-1 Nef protein as bait. Thereby, 21 novel cellular Nef interacting candidate proteins were uncovered. They are mostly integral membrane proteins located in various cellular compartments (Figure 5). The identified hits are involved in diverse cellular processes like apoptosis, neurogenesis, cell adhesion and protein quality control, and present a novel pool of host cell factors that might be affected by HIV-1 Nef.

### Comparison with the Results of Proteomic and RNAi Knockdown Studies

In general, Y2H approaches work best to identify direct bait-prey interactions, because exactly one prey protein is expressed with one bait protein within the same yeast cell. In contrast, mass spectrometry (MS) based approaches often yield proteins as hits that did not necessarily bind directly to the bait but are part of multi-protein complexes that bound to the bait. In addition, because the various prey proteins are not equally expressed in their native environment, MS based studies experience problems to identify proteins as positive hits when they are expressed at very low levels. Thus, it is obvious that Y2H screens and MS based approaches have different advantages and disadvantages and therefore do not necessarily yield the same set of positive hits, but render them rather complementary.

Concerning the very special case of HIV Nef, the situation becomes even more complex. In parallel with Nef’s pleiotropic functions, a cohort of cellular proteins has been described to interact with Nef. However, most of these interactions are considered to be low affinity interactions, and thus are not accessible with affinity purification approaches followed by MS analysis of the co-purified products. Such transient interactions are more easily detected by using a split-ubiquitin based Y2H screen, as was used in the present study. Here, Nef-prey complexes do not have to overcome lysis and purification steps, but only have to exist long enough to allow the ubiquitin hydrolase to separate the transactivating moiety from the Nef-prey complex, which subsequently switches on reporter gene transcription.

This may be an explanation, why our results did not yield any overlapping hit with the results from two recent MS based studies. Aiming specifically to understand Nef interactions with the cellular trafficking machinery, Mukerji et al. identified ten cellular Nef binders (namely EXOC1, EXOC2, EXOC3, EXOC4, EXOC6, NFKB1, PAK2, Q9NP29, Q9UL85, RPS20), which are suggested to be involved in Pak2-association dependent Nef functions [25]. In a more global approach carried out by Jäger et al., only four cellular proteins ACOT8, NMT1, MTDC and EXOC4 have been identified as Nef binding candidates [26] with EXOC4 being the only overlapping hit between both studies.

A third recently reported MS based study searched for cellular proteins that bind to a short peptide including the Nef secretion modification region (SMR) and identified four SMR-interacting cellular proteins [27]. The most abundant one was identified to be HSPA9/mortalin. Consistently, mortalin was coimmunoprecipitated with Nef-GFP from Jurkat T cells, and further studies (e.g. SMR-peptide inhibition assays, miRNA knockdown, antibody inhibition) suggested that mortalin possibly delivers Nef to endocytic vesicles, which then are released as exosomes. Notably, in our Y2H-based screen, we independently identified HSPA9/mortalin as a binding partner of membrane-anchored Nef as well (see Table S1). Please note that mortalin belongs to the few cytosolic proteins that were identified during our screen. Although we identified only one common hit with this MS based study, it is an important proof for the validity and specificity of our Y2H based screen in general.

Several recent genome-wide screening approaches for reduced viral replication upon RNAi knock down revealed more than 1000 annotated gene candidates for cellular proteins, also called “HIV-1 dependency factors” (HDF) that negatively influenced viral replication in cell culture [28]–[31]. Although hits of such RNAi screens cannot be assigned to specific HIV-1 proteins, as for example Nef, and it is well known that Nef is dispensable for HIV replication in cell cultures [32], which actually was the readout in the mentioned RNAi screens, we compared our hits with the lists of the published HDFs. Not very surprisingly, only one common hit was found with the study of Yeung et al. [30], namely PMEPA1/TMEPAI (see Table S1).

Based on known facts in literature, in the following we discuss the potential of selected hits, namely GPM6B, BAP31, CD320/TCblR and CLDN10, to be involved in HIV-1 pathogenesis. This includes the hits that were investigated further in this study.

### Glycoprotein M6B (GPM6B)

The glycoprotein M6B (GPM6B) has been the most prominent hit within the screen and like the glycoprotein M6A (GPM6A), is an integral membrane protein with four transmembrane domains. GPM6B is thought to have a function in the development of the nervous system [33]. GPM6B was firstly described as a brain specific protein expressed mainly in neurons and oligodendrocytes [34], but several recent studies demonstrate its broad distribution throughout many cell types and tissues [17], [35], [36]. HIV-1 infects microglial cells and astrocytes in the brain and an evidence of Nef being present in other cell type inside the CNS is insufficient to date. Also in recent literature, Nef has been reported to be translocated from HIV-1 infected cells to bystander cells via secretion, trogocytosis or nanotubes [37]–[39] and it is conceivable that Nef might be also secreted in the CNS from microglial cells and astrocytes and thereby might indirectly affect neurons or oligodendrocytes. Taken together, a clash of Nef and GPM6B seems to be possible in many cell types, but clearly further experiments would be necessary to prove this.

Our *in vitro* binding experiments using synthetic peptides, comprising the putative Nef binding region of GPM6B and recombinant Nef resulted in a robust binding with K*_D_*-values below 1 µM. We demonstrated that Nef negatively influences a function of GPM6B, namely the GPM6B-mediated induction of membrane extensions in Cos-7 cells, possibly as a result of a Nef-mediated downregulation of GPM6B. GPM6B-mediated membrane extensions have recently been reported for hippocampal neurons, where filopodia formation by GPM6B was analyzed and characterized as a stress-mediated regulator of hippocampal development. Similar effects were also described for other cells types overexpressing GPM6B, in particular also for Cos-7 cell lines, the cell type we used for our study [16].

Fjorback et al. described an interaction of GPM6B with the serotonin transporter (SERT) and a GPM6B-modulated uptake of serotonin [40]. Interestingly, with the serotonin receptor (5-HT4) Yeung et al. identified another compound of the serotonin uptake machinery as HDF [30]. These findings can help to unravel the mechanisms underlying the benefit of drugs acting as serotonin reuptake inhibitors [41], [42] or serotonin receptor antagonist [43], [44] observed for HIV-1 infected patients.

### B-cell Receptor-associated Protein 31 (BAP31)

B-cell receptor-associated protein 31 (BAP31) was identified ten times and is an ubiquitous protein residing in the endoplasmic reticulum (ER) of 28 kDa, forming a multi-pass membrane protein with three transmembrane domains, which can be processed to p20 BAP31 by caspase 8 cleavage [45]. BAP31 is known to be involved in the transport of nascent proteins at the ER membrane and presents an important factor in the quality control within the ER-Golgi intermediate compartment (ERGIC) and ER-associated degradation (ERAD) pathway [46]. Remarkably, certain viruses, hijack the ERAD pathway to evade the immune system or to enter the cytosol respectively [47]–[51].

Amongst the most described cargo of BAP31 is the major histocompatibility complex (MHC or human HLA) class I molecule [52]. Again, viruses have evolved elegant strategies to inhibit various stages of the MHC I antigen presentation pathway and thus evade a cellular surveillance mechanism (for review, see [53]). HIV-1 Nef downregulates MHC I surface expression, both by recruiting MHC I directly from the cell surface and by transporting newly synthesized MHC I molecules to lysosomes (for review see [54]). An important step during this process is the Nef mediated association of the cytoplasmic domain of MHC I with the clathrin adaptor protein complex 1 (AP1) at the trans-Golgi network, which recently could be solved at atomic resolution [55]. Beside this, several observations suggest that Nef also might interact with MHC I molecules earlier during the secretory pathway ([56], [57]). A direct interaction of Nef with BAP31 would support these findings and may imply that Nef additionally could interfere with the MHC I molecules upstream of the trans-Golgi network.

To date, BAP31 has been reported only once to be directly bound by a viral protein, namely the E5 protein of the human papillomavirus [58].

### CD320 Molecule/Transcobalamin Receptor (TCblR)

The CD320 molecule as a clearly Nef dependent hit was identified twice during the Y2H screen. CD320 was first thought to be specific for human B cells and in follicular dendritic cells [59]. The function of CD320 as transcobalamin receptor (TCblR) was discovered recently by Quadros et al. [60]. CD320/TCblR is a plasma membrane resident protein and mediates the uptake of extracellular vitamin B_12_ also known as cobalamin (Cbl) in a transcobalamin-bound manner. Like for the PLP proteins, we recognized a similarity to the known binding motif of Nef to the human CD4 molecule also inside the short cytoplasmic tail of CD320/TCblR.

### Claudin 10 (CLDN10)

For claudin 10 (CLDN10), most of the literature to date shows an involvement in cancer progression, especially of hepatocellular carcinoma [18], [61], [62]. However, high CLDN10 mRNA expression levels have been observed in mouse brain capillary endothelial cells. Therefore, a role of CLDN10 in tight junction formation at the blood-brain barrier (BBB) has been postulated, [63]. Accordingly, viruses make use of claudins to disrupt the BBB and to traverse epithelial cell layers. Known targets are for example claudin 1, claudin 6 and claudin 9, which have been identified as coreceptors for hepatitis C virus (HCV) entry [64]. Claudin 7 is involved in the infection of CD4(-) cells through HIV-1 [65] and the HIV-1 Tat protein is held to be responsible for an increased permeability of the BBB by influencing claudins [66], [67]. Possibly, disturbance of the BBB could be enhanced by an interaction of Nef and CLDN10.

Overall, the above discussed four proteins are only one fifth of the newly identified proteins that are prone to be involved in a direct interaction with Nef inside the host cell. Except for HSPA9/mortalin [27], all of the identified proteins are reported here for the first time to be Nef binding partners and these results may help to further understand the molecular mechanisms of Nef during HIV pathogenesis. We carried out independent experiments, including *in vitro* binding assays or confocal microscopy, with a subset of the newly identified putative Nef targeted host proteins and confirmed their interaction with Nef.

It is hard to imagine that all of the hereby revealed Nef interactions are relevant for the HIV-1 infection cycle, but they are potentially relevant for the pathology of HIV infection especially at late stages of an HIV infection. One of such collateral damages of HIV-infection may be the HIV-associated dementia.

### Conclusions

Our approach to identify Nef binding proteins yielded 21 human proteins, of which approx. 80% were membrane proteins, which underlines the special features of the employed split-ubiquitin Y2H screening method. For BAP31 and CLDN10 we demonstrated colocalization. For GPM6B and CD320, we additionally characterized binding motifs that are conserved for Nef binding to human CD4. Putative biological relevance of selected interactions is discussed. The results will contribute towards a better understanding of HIV-1 pathology.

## Methods

### Antibodies

Mouse monoclonal antibody directed against LexA was obtained from Santa Cruz Biotechnology. Anti-HA rabbit antibody (H 6908) was purchased from Sigma-Aldrich. Peroxidase conjugated ImmunoPure goat anti-mouse IgG (H+L) and ImmunoPure goat anti-rabbit IgG (H+L) were both from Pierce.

The following primary antibodies were used for immunostaining: anti-M6B rabbit polyclonal antibody (HPA002913, Sigma-Aldrich), goat polyclonal anti-BAP31 (sc-18579, Santa Cruz Biotechnology), goat polyclonal anti-CD320/TCblR (AF1557, R&D Systems) and mouse monoclonal anti-CLDN10 (BO1, Abnova). Polyclonal anti-Nef from rabbit (SA6102, Eurogentec) was produced from recombinant HIV-1 SF2 Nef_2–210_ as described previously [68].

### Yeast Two-hybrid Screen

The DUALhunter system (Dualsystems Biotech AG, Zurich, Switzerland), which is based on the split-ubiquitin system [69], was used to identify proteins that interact with membrane anchored HIV-1 Nef. The *Nef* gene from HIV-1 isolate SF2 was amplified from pUbi-Nef_2–210_ [68] and subcloned into the *Sfi*I sites of pDHB1. The resulting protein is a fusion of the small membrane anchor of Ost4p, Nef, residues 39 to 76 of yeast ubiquitin (Cub) and the LexA-VP16 transcription factor (Figure 1A). The pDHB1-Nef bait construct was transformed into the yeast strain NMY51. Absence of self-activation was confirmed by cotransformation or mating assays of the pDHB1-Nef bait, together with a control prey (pAI-Alg5 and pDL2-Alg5) and selection on minimal medium, lacking the amino acids tryptophane, leucine and histidine (selective medium) according to the manufactures instructions. To optimize the screening stringency for pDHB1-Nef, pilot screens were carried out according to the supplier instructions (DUALhunter kit user manual), in which the concentration of 3-aminotriazole (3-AT) – a competitive inhibitor of the HIS3 gene product – was adjusted. Thereby the library vector (pPR3-N), without an insert in place of a cDNA library, was transformed into the Nef bait-bearing strain. For the Y2H screen, pDHB1-Nef was cotransformed with a human adult brain cDNA library (DUALsystems Biotech AG), cloned in pPR3-N expressing the cDNAs as fusions carboxy-terminally of residues 1 to 38 of yeast ubiquitin (Nub) and an HA-tag into yeast strain NMY51. Transformants that were able to grow on selective medium were tested for their β-galactosidase activity using the filter lift-off assay (described in User manual DUALhunter kit, DUALsystems). More than 99% of the tested transformants showed β-galactosidase activity and were considered to be positives. Library plasmids were isolated from the positive clones, sequenced and analyzed.

### Bait Dependency Test

The respective pPR3 prey vector DNAs of each of the hits was isolated and retransformed into yeast strain NMY51 either with the bait plasmid (pDHB1-Nef) or with a control bait (pDHB1-largeT) encoding an Ost4p-largeT-Cub-LexA-VP16- fusion. Retransformants were analyzed for their growth and phenotype on plates lacking leucine and tryptophane (SD -leu -trp) or on plates additionally lacking histidine and adenine (SD -leu -trp -his -ade) as well as for their β-galactosidase activity as described in more detail in the DUALhunter kit handbook (DUALsystems). Only those who showed a prominent β-galactosidase activity when coexpressed with the Nef-bait, but not when coexpressed with the largeT control bait, were considered to be Nef dependent positive ligands.

### Plasmid Constructs

The Nef coding sequence was amplified from pUbi-Nef [68] without the stop codon, using sense and antisense primers including unique *Eco*RI and *Bam*HI sites. The PCR product was subcloned in-frame into the respective sites of pDsRed-N1 vector (Clontech), yielding pNef-DsRed coding for a Nef protein N-terminally fused to DsRed.

All potential interaction partners of Nef were subcloned from the Y2H screen vector pPR3-N via *Sfi*I sites into pHA-Mex-eGFP vector (DUALSystems) yielding pHA-Mex-eGFP-GPM6B, pHA-Mex-eGFP-BAP31, pHA-Mex-eGFP-CD320 and pHA-Mex-eGFP-CLDN10 coding for hit proteins C-terminally fused to eGFP, henceforth only named pGFP-hit for convenience. Lower case letters mark the amino acid positions of the used protein regions. Numbering is done according to the database entries (UniProtKB), which are P51572, Q9NPF0, P78369 and Q13491 for BAP31, CD320/TCblR, CLDN10 and GPM6B respectively.

### Immunoprecipitation Analysis

*S. cerevisiae* cells were cotransformed with the prey expression plasmid (pPR3-N-prey), including a HA-tag and either a Nef bait expression plasmid (pDHB1-Nef) or a control LargeT bait expression plasmid (pDHB1-LargeT) both including the LexA encoding region. Cells were lysed by vortexing with glass beads in 50 mM HEPES pH 7.4, 50 mM NaCl, 1% Triton X-100 and a cocktail of protease inhibitors (Complete Mini EDTA-free, Roche). Glass beads were separated from the lysate by low speed centrifugation and the resulting supernatant was centrifuged at higher speed (20 sec and 14.000 rpm). Membranes were separated from the remaining supernatant by centrifugation at 48.000 rpm for 30 min and 4°C, and subsequently lysed in 120 µl Triton buffer (50 mM TrisHCl pH 7.5, 150 mM NaCl, 0.3 mM MgCl_2_, 0.5% Triton X-100, 10% glycerin and a cocktail of protease inhibitors). The resulting extracts were subjected to immunoprecipitation with anti-HA or anti-LexA antibody. Immunoprecipitates were separated by SDS-PAGE and probed by western blot analysis for HA or LexA immunoreactivity. Nef-LexA (70 kDa) and largeT-LexA (114 kDa) immunoreactivity was detected with mouse monoclonal antibody directed against the LexA domain, and a HRP-conjugated goat anti-mouse IgG. Prey proteins immunreactivity was detected with rabbit anti-HA antibody, and a HRP-conjugated goat anti-rabbit IgG.

### Western Blots

Equal amounts of the respective samples from the CoIP were subjected to denaturing electrophoresis on a 12% sodium dodecyl sulfate-polyacrylamide gel. Proteins were transferred to a polyvinylidene difluoride membrane (10 mA, 40 min) which was blocked by 10% BSA (Sigma) for 30 min. The membrane was incubated with specific primary antibody overnight, followed by incubation with peroxidase conjugated secondary antibody for 90 min. Blots were visualized by chemiluminescence (SuperSignal West Pico Chemiluminescent Substrate, Pierce) and documented using the ChemiDoc system (Bio-Rad).

### Cell Culture

The Cos-7 (African green monkey kidney) cell line was obtained from DSMZ and cultivated in Dulbecco’s modified Eagle’s medium (DMEM) with 4.5 g/l glucose containing 10% fetal bovine serum at 37°C and in a 5% CO_2_ atmosphere. At confluence between 80 and 90% cells were subcultivated and counted using a coverslipped improved Neubauer chamber. Cells were passaged with Trypsin/EDTA and seeded at a density of 2×10^6^ cells per 75 m^2^ flask and used between passages 10 and 25 for all experiments. Furthermore they were regularly tested for mycoplasm contamination.

### Transfection

Transient transfection of Cos-7 cells was performed with the electroporator GenePulser (Biorad) set to an exponential pulse, 450 µF and 450 V in a 4 mm cuvette. Generally, 1×10^6^ cells/ml were used per transfection and plated out in 10x35 mm dishes with glass cover slides. Per transfection, a total amount of 10 µg DNA was used (endofree, QIAGEN EndoFree Plasmid Purification Maxi kit). After transfection, cells were incubated at 24 h.

### Immunocytochemical Staining

Cells were fixed either with 4% paraformaldehyde or with methanol:acetone (1∶1) for 1 min, then permeabilized with 0.2% (v/v) Triton X-100/PBS for 10 min followed by blocking with 1% BSA in PBS for 30 min up to 1 h. Primary antibodies were diluted 1∶500 in blocking buffer. Secondary antibody conjugated with Alexa488, Alexa647 or Cy5 were used at a 1∶500 dilution. DAPI staining of the nucleus was performed before the cover slips were mounted with fluorescent mounting medium (Trevigen) on glass slides and sealed.

### Microscopy

Microscopy was done with a confocal laser scanning microscope (LSM 710, Zeiss MicroImaging Inc.) equipped with an EC-Plan-Neofluar 40x/1.30 oil DIC objective. Images were acquired using Zen 2008 Software from Zeiss. Pixel dwell was set to 3.15 µs, image size 1024x1024, 8-bit, averaging over 4 times. Experiments were repeated independently at least twice and cells of minimum 5 fields of view were taken.

## Supporting Information

Table S1 Overview of the identified hits from the Y2H-screen with membrane-associated HIV-1 Nef. The table summarizes the characteristics of the identified positive interactors via DUALhunter system. Information is given for the complete protein name, synonyms, subcellular localization, main known function, tissue specify as well as its involvement in disease, if known. The information for each putative interaction partner of membrane-bound Nef was evaluated on the basis of the gene data bank (NCBI gene) and protein knowledgebase bank (UniProtKB) annotations.
(DOCX)
