# Supplementary material for: Brain Transcriptome-Wide Screen for HIV-1 Nef Protein Interaction Partners Reveals Various Membrane-Associated Proteins
Source: PLoS One. 2012 Dec 17;7(12):e51578. doi: 10.1371/journal.pone.0051578 (PMC3524239; doi:10.1371/journal.pone.0051578)
Supplement: Table S1 — Overview of the identified hits from the Y2H-screen with membrane-associated HIV-1 Nef. The table summarizes the characteristics of the identified positive interactors via DUALhunter system. Information is given for the complete protein name, synonyms, subcellular localization, main known function, tissue specify as well as its involvement in disease, if known. The information for each putative interaction partner of membrane-bound Nef was evaluated on the basis of the gene data bank (NCBI gene) and protein knowledgebase bank (UniProtKB) annotations. (DOCX) [file pone.0051578.s001.docx]

| Gene Symbol | Name | N° of hits | Gene ID | Synonyms | Subcellular location | function | tissue specifity | disease |
| --- | --- | --- | --- | --- | --- | --- | --- | --- |
| GPM6B | Neuronal membrane glycoprotein M6B | **23** | 2824 | M6B | integral membrane | proteolipid protein family; cellular housekeeping functions (trafficking and cell-to-cell communication); nervous system development | neuron, most brain regions |  |
| BAP31 | B-cell receptor-associated protein 31 | **10** | 10134 | BCAP31, DXS1357E | plasma membrane, ER and Golgi membrane, cytosol | transport of membrane proteins in ER to Golgi (ER-quality control compartment); caspase 8-mediated apoptosis | ubiquitous | BAP31 is deleted in the chromosome Xq28 deletion syndrome |
| CD320 | CD320 molecule, Transcobalamin receptor | **2** | 51293 | TCblR, 8D6A | cell surface; ER; integral membrane | transcobalamin receptor mediates VitaminB12 uptake; B-cell proliferation and immunoglobin secretion |  | methylmalonic aciduria type TCblR (MMATC) |
| CYB5B | Cytochrome b5 type B | **2** | 80777 | CYB5M, OMB5 | mitochondrion outer membrane | cytochrome b5 functions as electron carrier | lymphoma prostate cancer |  |
| GPM6A | Neuronal membrane glycoprotein M6A | **2** | 2823 | M6A | integral membrane; cell surface | calcium ion transmembrane transport |  |  |
| TSPAN7 | Tetraspanin 7 | **2** | 7102 | A15, DXS1692E, MXS1, TM4SF2 | integral plasma membrane, cell-surface protein | tetraspanin family; glycoprotein; signal transduction in cell development activation, growth, motility; neurite outgrowth | T cell, myelotic leukemia cells | X-linked mental retardation and neuropsychiatric disease e.g. Huntington's chorea |
| APR-3 | Apoptosis-related protein 3 | **1** | 51374 | C2orf28 | plasma membrane | apoptosis; hematopoietic development and differentiation | hematopoietic cell lines; upregulated in several tumor cell lines |  |
| B3GNT1 | N-acetyllactosaminide beta-1,3-N-acetylglucosaminyl-transferase | **1** | 11041 | B3GNT6 | Golgi apparatus membrane; integral membrane | catalytic activity; protein modification + glycosylation; synthesis or the elongation of the linear poly-N-acetyl-lactosaminoglycans | heart, brain, skeletal muscle and kidney and to a lesser extent in placenta, pancreas, spleen, prostate, testis, ovary, small intestine and colon. |  |
| CHN1 | Chimerin 1 | **1** | 1123 | ARHGAP2, CHN | cytosol | GTPase-activating protein; neuronal signal-transduction mechanisms | neurons | DURS2 syndrome (motility disorder; retraction syndrome) |
| CLDN10 | Claudin 10 | **1** | 9071 |  | membrane cell junction | tight junction; calcium-independent cell-adhesion activity | Liver, skeletal muscle | primary hepatocellular carcinoma, cervix carcinoma |
| HSPA9 | Heat shock 70kDa protein 9 | **1** | 3313 | mortalin | mitochondrion, ER, plasma membrane; cytoplasmic vesicles, nucleus | cell proliferation; cellular aging; may act as chaperone; ATP-binding; import nuclear-encoded proteins to mitochondria |  | mutations of HSPA9 higher risk of Parkinson's disease; Mortalin regulated by APOE in AD |
| ITGB1 | Integrin, beta 1 | **1** | 3688 | FNRB, MDF2, MSK12 | plasma membrane, cell surface | membrane receptor; cell adhesion; embryogenisis, hemostasis, tissue repair; immune response | Isoform beta-1A widely expressed, other isoforms coexpressed with restricted distribution | seems to enhance angiogenesis in Kaposi's sarcoma lesions by an interaction with extracellular HIV-1 Tat protein |
| OCIAD1 | OCIA domain containing 1 | **1** | 54940 | OCIA | endosome, mitochondion | ovarian cancer recurrence, tumour metastasis | ISO*1: testis, brain, placenta, ovary, prostate, mammary gland; ISO2 CNS, brain, cerebellum, spinal cord |  |
| PEBP1 | Phosphatidyl-ethanolamine binding protein 1 | **1** | 5037 | PBP, PEBP | cytoplasm, rER golgi, cell surface, mitochondrial outer membrane, | ATP binding; mitogen-activated protein kinase; brain development; involved in acetylcholine, cAMP and MAPKKK processes | neuron, synaptosome; placenta, brain, lung, liver… | adenocarcinoma |
| PH4 | Hypoxia-inducible factor (HIF) prolyl 4-hydroxylase | **1** | 54681 | P4HTM | transmembrane ER | calcium and iron ion binding, oxidoreductase activity (cellular oxigen sensor) | adult pancreas, heart, skeletal muscle, brain, placenta, kidney and adrenal gland; fibroblasts |  |
| PMEPA1 | Transmembrane prostate androgen-induced protein | **1** | 56937 | STAG1, TMEPAI | integral membrane, plasma membrane | androgen receptor signaling pathway, NEDD4 mediated interaction | prostate, ovary | inflamatory pathways in digestive cancers |
| VSIG4 | V-set and immunoglobulin domain containing 4 | **1** | 11326 | CRIg, Z39IG | integral membrane | negative regulation of T cell proliferation; interleucin protcution inhibitor | resting macrophage, lung, placenta and fetal tissues (abundantly) |  |

*ISO = isoform
